# Asymmetric Mach–Zehnder Interferometric Biosensing for Quantitative and Sensitive Multiplex Detection of Anti-SARS-CoV-2 Antibodies in Human Plasma

**DOI:** 10.3390/bios12080553

**Published:** 2022-07-22

**Authors:** Geert Besselink, Anke Schütz-Trilling, Janneke Veerbeek, Michelle Verbruggen, Adriaan van der Meer, Rens Schonenberg, Henk Dam, Kevin Evers, Ernst Lindhout, Anja Garritsen, Aart van Amerongen, Wout Knoben, Luc Scheres

**Affiliations:** 1 Surfix Diagnostics, Plus Ultra Building, Bronland 12 B-1, 6708 WH Wageningen, The Netherlands; anke.trilling@surfixdx.com (A.S.-T.); janneke.veerbeek@surfixdx.com (J.V.); michelle.verbruggen@surfixdx.com (M.V.); adriaan.vandermeer@surfixdx.com (A.v.d.M.); rens.schonenberg@surfixdx.com (R.S.); henk.dam@surfixdx.com (H.D.); kevin.evers@surfixdx.com (K.E.); wout.knoben@surfixdx.com (W.K.); luc.scheres@surfixdx.com (L.S.); 2 Future Diagnostics Solutions, Nieuweweg 279, 6603 BN Wijchen, The Netherlands; lindhout.e@future-diagnostics.nl; 3 Innatoss Laboratories B.V., Kloosterstraat 9, 5349 AB Oss, The Netherlands; anja.garritsen@innatoss.com; 4 Wageningen Food & Biobased Research, Bornse Weilanden 9, 6708 WG Wageningen, The Netherlands; aart.vanamerongen@wur.nl

**Keywords:** biosensor, photonics, SARS-CoV-2, antibodies, diagnostics, serology

## Abstract

The Severe Acute Respiratory Syndrome Coronavirus 2 (SARS-CoV-2) pandemic has once more emphasized the urgent need for accurate and fast point-of-care (POC) diagnostics for outbreak control and prevention. The main challenge in the development of POC in vitro diagnostics (IVD) is to combine a short time to result with a high sensitivity, and to keep the testing cost-effective. In this respect, sensors based on photonic integrated circuits (PICs) may offer advantages as they have features such as a high analytical sensitivity, capability for multiplexing, ease of miniaturization, and the potential for high-volume manufacturing. One special type of PIC sensor is the asymmetric Mach–Zehnder Interferometer (aMZI), which is characterized by a high and tunable analytical sensitivity. The current work describes the application of an aMZI-based biosensor platform for sensitive and multiplex detection of anti-SARS-CoV-2 antibodies in human plasma samples using the spike protein (SP), the receptor-binding domain (RBD), and the nucleocapsid protein (NP) as target antigens. The results are in good agreement with several CE-IVD marked reference methods and demonstrate the potential of the aMZI biosensor technology for further development into a photonic IVD platform.

## 1. Introduction

Biosensors are valuable tools in a wide range of application areas such as medical diagnostics, agri-food, and environmental monitoring [1,2]. Application of biosensors in point-of-care (POC) in vitro diagnostics (IVD) is promising as they may fulfill the need for sensitive, robust, and cost-effective POC testing platforms for disease diagnosis outside of the lab. Early diagnosis and effective treatment enabled by POC diagnostics support a more efficient and patient-centered healthcare system. Moreover, the availability of affordable POC diagnostics is of crucial importance for developing countries, where resources and access to healthcare facilities are limited [3]. The Severe Acute Respiratory Syndrome Coronavirus 2 (SARS-CoV-2) pandemic has once more emphasized the value of and the urgent need for accurate and fast POC diagnostics for outbreak control and prevention [4,5,6].

Infection with SARS-CoV-2 induces an immune response in the host that normally results in the generation of different isotype antibodies (IgM, IgG, and IgA) against specific viral antigens such as the spike protein (SP), the receptor-binding domain (RBD), and the nucleocapsid protein (NP) [7]. Seroconversion for IgG typically takes about two to three weeks after symptoms onset while IgG antibody waning typically sets in after two to three months [8,9]. Serological testing is useful for confirming if individual cases have been infected in the past, for assessing seroprevalence and overall exposure of the host population [10], and for assessing vaccination response and efficiency. 

Various IVD tests for SARS-CoV-2 specific antibodies are being used in the laboratory. Up till now, worldwide, 356 IVD registered serology tests are available, of which 219 are CE-IVD marked and 73 have obtained the FDA Emergency Use Authorization (EUA) [11]. Most of these tests involve enzyme-linked immunosorbent assay (ELISA), chemiluminescent immunoassay (CLIA), and lateral flow assay (LFA) test formats. Each test format has its own advantages and disadvantages: ELISA is a well-known and proven quantitative test, but the method is laborious and time-consuming and has to be performed by skilled personnel. CLIA is a highly automated and high-throughput quantitative method, but mostly depends on bulky and expensive measurement platforms. LFA is fast, simple, and relatively inexpensive, which explains why testing with lateral flow test strips has become rather customary for POC applications. However, LFA is not a quantitative test and may have a lower performance, especially with regard to sensitivity [12].

To bridge the gap between POC testing with LFA and remote (clinical lab) testing with ELISA or CLIA, several alternative POC testing formats are being developed such as lab-on-a-chip (LOC) and lab-on-a-disc (LOAD) [4,13,14,15]. Integration of microfluidics in the POC device might be beneficial as it offers possibilities for improving compactness and limiting reagent consumption and might also help in further reducing the amount of required patient sample [16]. To improve POC biomarker detection, different types of sensitive and real-time measuring biosensors have been suggested for implementation in POC devices [5,17,18,19] such as electrochemical sensors and optical sensors based on surface plasmon resonance (SPR), surface-enhanced Raman scattering (SERS), fluorescence, and chemiluminescence. Photonic biosensing technologies that have been explored for the possible use in SARS-CoV-2 serology include SPR [20,21,22,23,24], biolayer interferometry (BLI) [25], and microring resonators (MRRs) [26,27]. The last mentioned is a member of a special group of sensors called the photonic integrated circuit (PIC) biosensors.

PIC biosensors offer advantageous features such as a high analytical sensitivity, the capability for multiplexing and miniaturization, and the suitability for integration in optofluidic devices [28,29]. Additionally, these sensors offer advantages such as the prospect of label-free detection, the possibility of real-time measurement, immunity to electromagnetic interference, and the high potential for integration with other (micro) components. In addition, the photonic chips are manufactured by standard complementary metal-oxide semiconductor (CMOS)-compatible fabrication techniques. This is important to reduce the cost price of the chips as CMOS technology is ideally suited for high-volume manufacturing. Many types of PIC sensors have been described in the literature such as MRRs, grating coupler devices, photonic crystals, and interferometric waveguide sensors [19].

Surfix Diagnostics has developed a photonic biosensor platform based on the asymmetric Mach–Zehnder Interferometer (aMZI), which is intended for generic, label-free, and sensitive multiplex detection of a wide variety of targets, such as proteins, DNA, RNA, viruses, bacteria, etc. A major advantage of the aMZI design is that the sensor sensitivity can be tailored by increasing the geometrical pathlength of the sensing arm and/or by decreasing the asymmetry of the aMZI (the difference in pathlength between the two interferometer arms) [30,31]. When combining the high intrinsic sensitivity of the aMZI sensor with Surfix’s proprietary material-selective surface modification, the analytical sensitivity can be even further enhanced [32]. In previous studies, aMZI-based biosensors have been used for the detection of food contaminants [33,34], ocean pollutants [35], streptavidin [36], and protein biomarkers for cancer [30]. While these studies show the potential and broad applicability of the technology, the amount of data presented was limited.

This paper describes the detection of different anti-SARS-CoV-2 specific antibodies (anti-SP, anti-RBD, and anti-NP) in a dilution series of an NIBSC-verified plasma calibrant in order to assess the analytical sensitivity and dynamic range of the photonic biosensor platform. Moreover, the diagnostic performance of the platform was evaluated by the detection of anti-SARS-CoV-2 specific antibodies in plasmas from a NIBSC verification panel. All results were compared to testing results obtained with several CE-IVD marked serology tests. Calibrant dilution tests showed a good limit of detection (LODs down to 0.3 IU/mL of calibrant) and dynamic ranges that were in accordance with most of the reference methods. The data obtained with the verification panel showed good scores for the Surfix photonic biosensor for distinguishing anti-SARS-CoV-2 antibody positive and anti-SARS-CoV-2 antibody negative plasmas.

In this explorative validation study, the performance of the Surfix photonic biosensor platform was successfully tested in a comparison with different CE-IVD marked reference methods, which demonstrates the potential of the method. Currently, efforts are directed at increasing the manufacturability of the system and developing it into an IVD platform for POC testing.

## 2. Materials and Methods

### 2.1. Materials

N-Hydroxysuccinimide (NHS), (1-ethyl-3-(3-dimethylaminopropyl)carbodiimide hydrochloride (EDC), trehalose, bovine serum albumin (BSA; heat shock fraction, ≥98%), sodium dodecyl sulfate solution (SDS, 10% in H_2_O), Tween 20, sodium chloride (NaCl), sodium hydroxide (NaOH), hydrochloric acid solution (1 M), phosphate-buffered saline (PBS) tablets (0.01 M phosphate buffer, 0.0027 M potassium chloride and 0.137 M sodium chloride, pH 7.4), sodium phosphate (dibasic dihydrate and monobasic monohydrate, respectively), bicine (≥99%), sodium carbonate, and sodium bicarbonate were purchased from Sigma-Aldrich (St. Louis, MO, USA). Methanol (≥99.5%) was obtained from VWR. 2-(N-morpholino)ethanesulfonic acid (MES hydrate, ≥99.5%) was obtained from Fluka. SARS-CoV-2 (2019-nCoV) Spike S1 + S2 ECD-His (referred to as SP; 40589-V08H4, host: HEK293 cells), SARS-CoV-2 Nucleocapsid-His SARS-CoV-2 (2019-nCoV) Nucleocapsid-His (referred to as NP; 40588-V07E, supplied in PBS, host: *E. coli*), and SARS-CoV-2 (2019-nCov) Spike RBD-His (referred to as RBD; 40592-V08H, supplied in PBS, host: HEK293 cells) recombinant proteins were obtained from Sino Biological Europe GmbH (Eschborn, Germany). Affinity-purified polyclonal rabbit anti-human IgG (Fcγ fragment-specific, 309-005-008) was obtained from Jackson ImmunoResearch (Cambridgeshire, UK). Affinity-purified polyclonal rabbit anti-nucleocapsid IgG (GTX135361) was obtained from GeneTex, Inc. (Irvine, CA, USA). Affinity-purified polyclonal goat anti-rabbit IgG (heavy&light chain-specific, Atto 488 labeled, ABIN964982) was purchased from antibodies-online GmbH (Aachen, Germany). Anti-SARS-CoV-2 antibody negative plasma panel (DSPA 4.9.11.1) was obtained from in.vent Diagnostica GmbH (Hennigsdorf, Germany). CE-marked material anti-SARS-CoV-2 verification panel for serology assays (20/B770-02) and Anti-SARS-CoV-2 antibody diagnostic calibrant reagent (20/162) were obtained from the National Institute for Biological Standards and Control (NIBSC, Potters Bar, UK). Ultrapure water (18.2 MΩ.cm at 25 °C) was prepared using the Puranity TU3 UV/UF+ system (VWR International).

### 2.2. Chip Design and Operation

**Figure 1:**
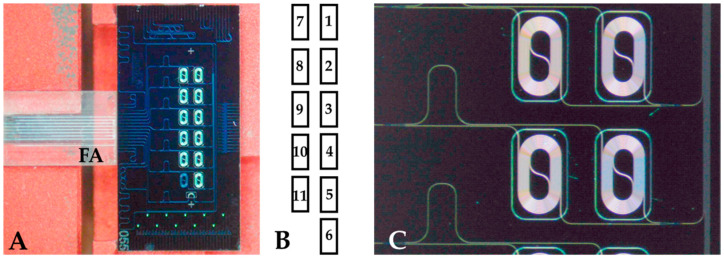
(**A**) Photomicrograph of the asymmetric Mach–Zehnder (aMZI) chip (dimensions: 5 × 10 mm) with an aligned Fiber Array (FA). (**B**) Assignment scheme for the five balanced and the one unbalanced aMZI biosensors regarding the spotting of viral antigens: 1, 4: spike protein (SP); 2: receptor binding domain (RBD); 3, 5: nucleocapsid protein (NP); 6–11: bovine serum albumin (BSA). (**C**) Zoom-in on two balanced aMZI sensors with a view of the waveguide spirals and sensing windows.

The heart of the system is the photonic chip, which contains an array of planar waveguide-based aMZI biosensors (Figure 1). The chips were fabricated by LioniX International (Enschede, The Netherlands). The photonic chips have dimensions of 10 mm × 5 mm and are based on a single stripe TriPleX™ geometry [37] containing a stoichiometric Si_3_N_4_ core (a height of 100 nm and a width of 1000 nm), on top of a 6 µm thermal SiO_2_ substrate, and 4 µm top cladding. The on-chip circuitry consists of a spot-size convertor (allowing for a very efficient fiber-to-chip light coupling) and a 1 × 8 splitter (based on subsequent Y-branches) in order to achieve an even distribution of the input light over the 8 individual aMZI sensor elements (6 aMZI biosensors and 2 auxiliary aMZI’s), with each aMZI sensor being connected to an individual output waveguide.

**Figure 2:**
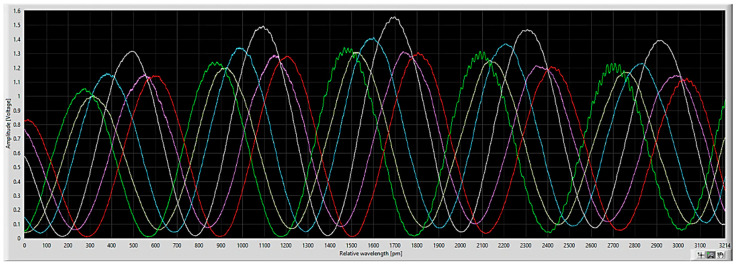
Example of a transmission spectrum overlay determined for the 6 different aMZI biosensors (*x*-axis: wavelength scan position (in picometer), *y*-axis: optical power output (in Volt)).

In-coupling and out-coupling of light was realized by butt-end coupling of fibers to the chip facet by means of an optimized fiber array (Figure 1A). The operating wavelength is about 850 nm, allowing the use of cost-effective and high-quality light sources (vertical-cavity surface-emitting lasers (VCSELs)) and detectors (photodiodes). A large part of the mode field of the light is confined to the waveguide core but a significant part propagates outside the waveguide. This component is called the evanescent field, which decays exponentially as a function of the distance to the waveguide surface (penetration depth ≈ 200 nm). At places where the sensor needs to interact with the sample, the SiO_2_ top cladding had been etched away locally (yielding the so-called sensing window) for exposing the Si_3_N_4_ waveguide to the sample or buffer solution. The specific binding of antibodies or other (bio)molecules onto the waveguide surface causes a local increase of the effective index of the propagating mode of the aMZI arm and, concomitantly, an increase of the optical pathlength. This leads to a phase shift in the sinusoidal optical power transfer function of the aMZI that can be measured as a shift in the transmission spectrum [30,33]. The transmission spectrum is continuously determined by measuring the optical power output of the aMZI as a function of the operating wavelength of the incoming light during wavelength scanning (Figure 2), which enables monitoring of the shift (in picometer) of the transmission spectrum. Wavelength scanning is performed by electrical modulation of the VCSEL at a frequency of 10 Hz (see Section 2.3).

Each aMZI biosensor contains two spiral-shaped sensor arms (a signal arm and a reference arm) with a geometrical pathlength of the waveguide within the sensing window of 12.5 mm (Figure 1C). The bulk sensitivity of each sensor is about 2000 nm per refractive index unit (RIU). Each chip contains five balanced biosensors and one unbalanced aMZI biosensor (Figure 1A,B). The term balanced means that both the signal arm and the reference arm are in direct contact with the sample or buffer solutions at the place of the sensing window (Figure 1C). Use of a reference arm that contacts the fluid has advantages as it allows for compensation for differences in bulk refractive index (as may exist, for example, between sample and run buffer) and is relatively insensitive to changes in temperature. The unbalanced aMZI (Figure 1B: sensor 6) has a signal arm that is in contact with the sample or buffer solutions and a reference arm that is covered with a SiO_2_ layer. This unbalanced aMZI was used to measure bulk refractive index changes in the liquid in order to monitor the injection and washing away of plasma sample.

### 2.3. Measurement Platform

The measurement platform consists of a liquid handling unit and an alignment stage (see below), and an optical signal read-out module (OSROM) [38]. The OSROM contains a tunable light source and 8 photodiode detectors, plus associated electronics and a built-in data acquisition (DAQ) unit (USB-6363, National Instruments, Austin, TX, USA). As a light source, a pigtailed VCSEL (type ULM850, TO46, 2.0 mW; obtained from Philips Technology GmbH, U-L-M Photonics, Ulm, Germany) with a nominal wavelength of 850 nm was used. The VCSEL is part of a thermally isolated compartment that is temperature controlled at about 40 °C (with a temperature stability of about 0.02 °C) by means of a Peltier element and a thermocouple. By varying the voltage/drive current over the VCSEL diode, a linear wavelength scan of the VCSEL light output is performed over a range of about 3 nm and at a frequency of 10 Hz, and simultaneously the optical power output of all 8 aMZI sensor elements is being monitored by means of the connected 8 photodiodes. The photodiode signals are amplified with low noise transimpedance amplifiers with adjustable gains. The obtained output is measured with the DAQ card enabling data transfer at 2 MB/s to the laptop.

The liquid handling unit contains components such as a peristaltic pump (P625/TS020P; Instech Laboratories, Inc., Plymouth Meeting, PA, USA), a syringe pump (NE-501; KF Technology, Rome, Italy), three automated stream selector valves (1 × 8, 1 × 6, and adapted load/inject valve; VICI AG, Schenkon, Switzerland) and associated polyether ether ketone (PEEK) tubing (inner diameter 0.01″, 0.02″ and 0.03″) with supply vials. All these components are brought together and connected to driving electronics and controlled by the software. Pumps and valves are operated via LabView based user-defined liquid handling scripts. Supply of liquid to the chip proceeds via a load/inject valve that is connected to a sample loop with an internal volume of 200 µL.

**Figure 3:**
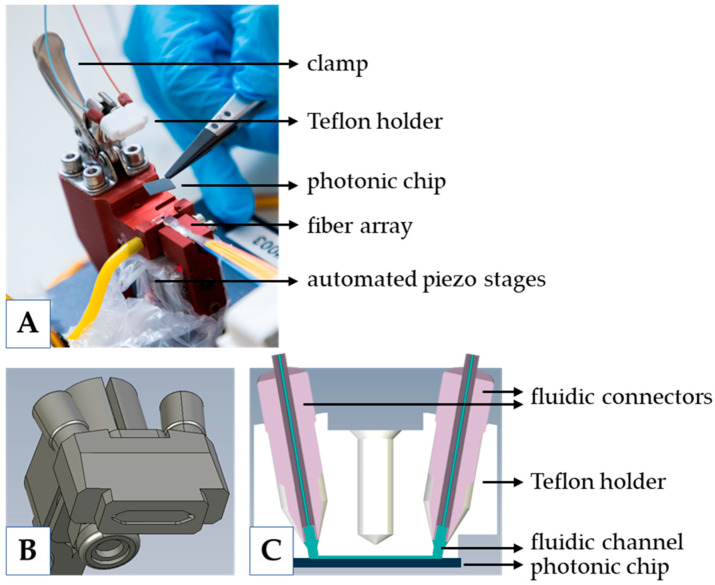
(**A**) Photograph of the alignment unit, where the chip is placed and brought in close contact with the fiber array. The flow cell is realized by clamping the Teflon holder on the chip. (**B**) Teflon holder close-up. (**C**) Cross-section of the Teflon holder on the chip, which defines the flow cell (liquid indicated in blue).

The photonic chip is placed on an alignment stage (Figure 3) to allow in-coupling and out-coupling of light, and to allow the flow of liquids over the chip. The alignment stage is custom designed and manufactured and has three main components: a frame that holds the chip, a clamp with a Teflon holder for the fluidic connection, and two piezoelectric linear stages with a control unit (12 mm travel, SLC; SmarAct GmbH, Oldenburg, Germany) to align the fiber array with respect to the chip (Figure 3).

### 2.4. Chip Functionalization

Functionalization of the chip surface was done by Surfix’s proprietary material-selective coating technology. This results in a carboxylate-terminated layer on the Si_3_N_4_ waveguide surface while the surrounding SiO_2_ surface is modified with a polyethylene glycol (PEG)-based antifouling layer. This way, bioreceptor immobilization and, consequently, analyte binding are confined exclusively to the waveguide surface, which leads to a higher analytical sensitivity and improved limit of detection [32].

Shortly before spotting of protein, the coated aMZI chip was sonicated in ultrapure water for 10 min and this step was repeated in methanol after which the chip was dried quickly by means of nitrogen blowing. Subsequently, a chemical activation of the carboxylate coating was performed by application on top of the chip of a 60 µL sessile drop of a freshly prepared EDC/NHS solution (0.2 M EDC and 0.1 M NHS in 10 mM MES pH 5.5) for 15 min. Then, after a quick rinse with ultrapure water and drying by means of nitrogen blowing, spotting was performed by means of the sciFLEXARRAYER S3 piezoelectric arrayer spotter (Scienion, Berlin, Germany). Spotting was performed with 4 different proteins according to the assignment shown in the caption of Figure 1B. The viral antigens were spotted at a concentration of 100 µg/mL in spotting buffer (for SP: 10 mM MES pH 5.3, for RBD: 10 mM bicine pH 9.0, for NP: 10 mM bicarbonate pH 9.8, all with 3% trehalose), while bovine serum albumin (BSA) was spotted in the MES spotting buffer at a concentration of 500 µg/mL. The spotted chips were incubated for 15 min and afterwards blocked by incubation with blocking buffer (PBS + 7% BSA) for 30 min. Finally, the chips were washed with buffer (PBS + 0.05% *v*/*v* Tween 20) for 5 min under gentle shaking followed by washing for 5 min with storage buffer (PBS + 3% trehalose) also under gentle shaking. The chips were stored in storage buffer at 4 °C for a maximum of three days until further use.

### 2.5. Plasma Measurements

Using the liquid handling system, the spotted photonic chips were conditioned for a maximum of 30 min at a flow of 15 µL/min with run buffer (PBS + 1% BSA + 0.05% *v*/*v* Tween 20). Then, sample was injected over the sensor surface for 10 min at a flow of 15 µL/min. For the NIBSC calibrant study, the calibrant (stock concentration 1000 IU/mL) was diluted with different amounts of anti-SARS-CoV-2 antibody negative plasma (pooled from three plasmas) resulting in calibrant concentrations of 0.25, 0.5, 1, 2, 5, 10, 20, 50, 100, 200, and 500 IU/mL. These different plasma mixtures were then diluted 10-fold with run buffer, resulting in 10% *v*/*v* plasma. The plasmas of the NIBSC verification panel were diluted 25-fold with run buffer, resulting in 4% *v*/*v* plasma. All samples from the NIBSC verification panel were measured in a blind manner, meaning that the operator did not know if the plasma was anti-SARS-CoV-2 positive or anti-SARS-CoV-2 negative. After plasma sample injection, the chips were washed with run buffer followed by an injection of secondary antibody (rabbit anti-human IgG, 24 µg/mL in run buffer) for 10 min at a flow of 15 µL/min. The resulting sensorgrams were recorded and from this, the total shifts in transmission spectrum (in picometer) that resulted from the incubation with human plasma sample and the incubation with secondary antibody, were determined. After each measurement, and before inserting a new aMZI chip, the liquid handling system was cleaned by flowing of 0.5% SDS solution for 6 min at a flow of 15 µL/min.

For comparison with the biosensor, plasma samples were also tested at Innatoss Laboratories (Oss, The Netherlands) with two commercial CE-IVD marked ELISAs: the Euroimmun SARS-CoV-2 IgG-S1 ELISA, which employs the S1-domain, and the Euroimmun SARS-CoV-2 IgG–NCP ELISA, modified to only contain diagnostically relevant epitopes of the NP antigen (Euroimmun, Lubeck, Germany). Furthermore, plasma samples were tested at Future Diagnostics (Wijchen, The Netherlands) with two commercial CE-IVD-marked CLIAs: the Abbott Architect SARS-CoV-2 IgG II assay, which uses the NP antigen (reference 06R8622, Abbott Laboratories Inc., Chicago, IL, USA), and the ADVIA Centaur SARS-CoV-2 IgG (sCOVG) assay, which uses the RBD domain (Siemens Centaur sCOVG assay, reference 11207376, Siemens Healthcare Diagnostics Inc., New York, NY, USA). All reference tests were performed in accordance with the manufacturer’s instructions.

For representation in dose-response curves, all data obtained on the calibrant were fitted by non-linear regression using a 4-parameter logistic (4PL) model (GraphPad Prism 9). Blank measurements were done in replicate (*n* = 10) on the anti-SARS-CoV-2 antibody negative plasma (pooled from three plasmas), used for diluting the calibrant, yielding values for each antigen (SP, RBD, NP) for both assay steps (incubation with plasma and with secondary antibody) from which the means and standard deviations were calculated.

## 3. Results

### 3.1. Material-Selective Sensor Modification

**Figure 4:**
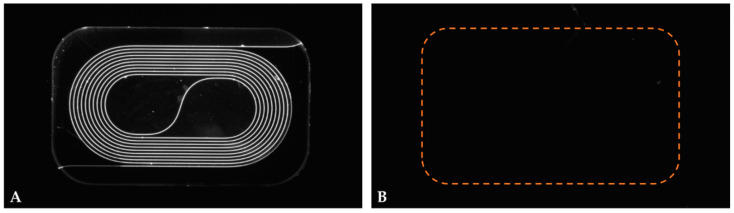
Fluorescence photomicrograph of (**A**) an NP-modified and (**B**) a BSA-modified spiral arm after incubation with polyclonal rabbit anti-NP antibody and subsequent incubation with Atto 488 labeled goat anti-rabbit IgG antibody. Note: the dotted orange line indicates the perimeter of the sensing window.

To demonstrate the effectiveness of the material-selective functionalization (a carboxylate-terminated layer on Si_3_N_4_ and a polyethylene glycol (PEG)-based layer on SiO_2_) for achieving selective immobilization of viral antigen to the Si_3_N_4_ waveguide surface, functionalized photonic chips were spotted with NP (viral antigen) and BSA (negative control protein). Next, the modified chips were incubated with rabbit anti-NP antibody followed by incubation with Atto 488 labeled goat anti-rabbit IgG. Finally, the chips were examined by means of fluorescence microscopy. As can be seen in Figure 4A, the resulting fluorescence was exclusively localized on the waveguide spiral indicating that modification of the sensor with NP antigen was confined to the carboxylate-terminated Si_3_N_4_ waveguide surface. In previous work, the added value of material-selective functionalization was demonstrated for improving the analytical sensitivity of a model system [32]. In addition, spiral arms modified with BSA (the negative control protein) did not show any fluorescence signal indicating a virtual absence of non-specific binding (Figure 4B).

### 3.2. Testing on Calibrant Dilution Series

To examine the dynamic range and analytical sensitivity of the six Surfix assays (direct and indirect detection of each of three antigens), a dilution series of anti-SARS-CoV-2 antibody plasma calibrant (NIBSC, 20/162) was measured. For comparison, the same dilution series was also measured by four CE-IVD-marked reference assays (two ELISA and two CLIA methods).

**Figure 5:**
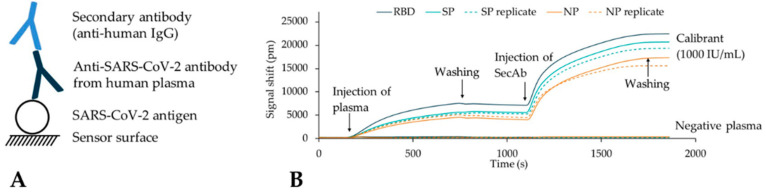
(**A**) Schematic representation of the binding complex that is formed during the assay. SARS-CoV-2 antigens (NP, RBD, and SP) are immobilized onto the sensor surface; during injection of plasma sample, SARS-CoV-2 specific antibodies (if present) bind to the antigens and, in turn, can be recognized and bound by secondary antibodies during the second incubation step. (**B**) Overlay of sensorgrams obtained for plasma calibrant (1000 IU/mL) and anti-SARS-CoV-2 antibody negative plasma.

Figure 5A shows a schematic of the binding complex that is formed on the biosensor surface illustrating the direct and indirect assay option. Also shown are sensorgrams (Figure 5B) obtained with the Surfix method on a negative control sample (10 times diluted negative plasma in buffer) and a calibrant sample (10 times diluted calibrant stock in buffer). Incubation with negative plasma did not lead to a shift in signal indicating the absence of non-specific binding. Incubation with calibrant plasma induced a large shift of the signal, which indicated binding of a plasma component, most probably IgG. Subsequent incubation with anti-human IgG secondary antibody also resulted in a substantial binding, confirming the identity of the previously bound component.

**Figure 6:**
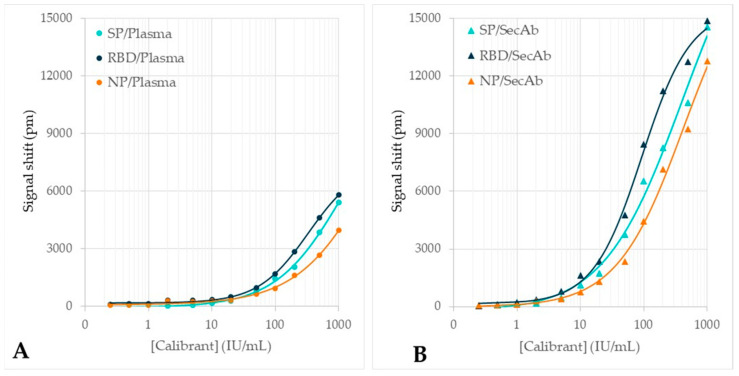
Dose-response curves obtained with the photonic biosensor when measuring the dilution series of plasma calibrant. Results are shown for (**A**) the incubation with diluted calibrant (direct assay) and (**B**) the incubation with anti-human IgG secondary antibody (indirect assay).

The dose-response curves of all Surfix assays as obtained on the calibrant dilution series (12 concentrations) are shown in Figure 6. For both the direct and the indirect assay, a difference between the antigens was found with an increase of the signal in the order NP < SP < RBD. Furthermore, the indirect assay showed an amplification with a factor of about three as compared to the signal obtained with the direct assay.

Replicate measurements (*n* = 10) were done on the anti-SARS-CoV-2 antibody negative pooled plasma while employing a new chip for each measurement. For each assay, the mean and standard deviation (SD) of the replicate measurements on negative pooled plasma were calculated and used to derive the limit of detection (LOD). The LOD was defined as the calibrant concentration at which the signal was equal to the mean plus three times the SD of the blank measurement (Table 1).

**Figure 7:**
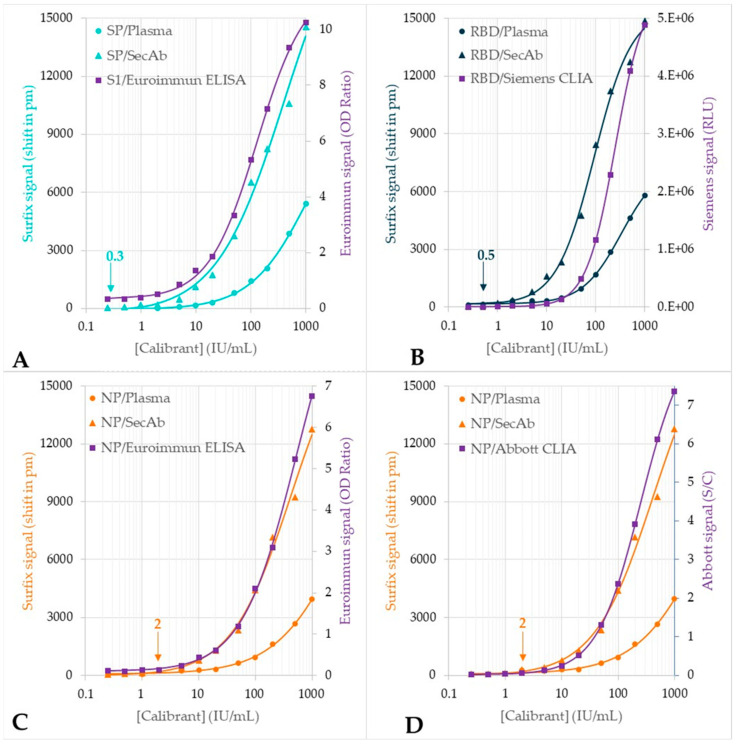
Dose-response curves for the plasma calibrant dilution series obtained with the photonic biosensor (*n* = 2) in a comparison with the outcome of the relevant reference test: (**A**) S1/reference test: Euroimmun SARS-CoV-2 IgG-S1 ELISA (IgG) (*n* = 3). (**B**) RBD/reference test: Siemens ADVIA Centaur SARS-CoV-2 IgG (sCOVG)) (*n* = 2). (**C**) NP/reference test: Euroimmun SARS-CoV-2 IgG-NCP ELISA (*n* = 3). (**D**) NP/reference test: Abbott Architect SARS-CoV-2 IgG (*n* = 2). Meaning of symbols: circles = plasma (direct assay), triangles = secondary antibody (indirect assay), squares: reference test. Note: the arrow near the x-axis indicates the LOD of each indirect Surfix assay.

The samples from the dilution series of the plasma calibrant were also tested with four CE-IVD-marked reference methods in order to validate the results from the photonic biosensor. Comparison of the dose-response curves generally shows a high resemblance between the Surfix indirect immunoassay and the reference tests (Figure 7).

The LODs of the Surfix indirect immunoassay (Table 1) are indicated in Figure 7 (arrow symbols near the x-axis) in order to mark the large dynamic range of the photonic biosensor, which is more than 3 decades. These LODs are comparable to the detection capability of the reference assays used in the comparison study.

**Figure 8:**
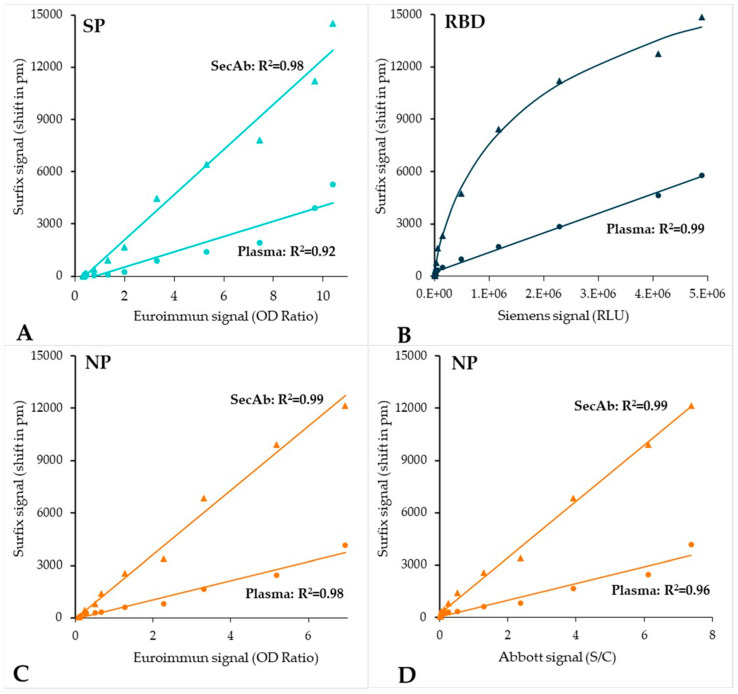
Correlation plots of the Surfix photonic biosensor results versus the results obtained with each of the reference methods: (**A**) Euroimmun SARS-CoV-2 IgG-S1 ELISA (IgG); (**B**) Siemens ADVIA Centaur^®^ SARS-CoV-2 IgG (sCOVG) CLIA; (**C**) Euroimmun SARS-CoV-2 IgG-NCP ELISA; (**D**) Abbott Architect SARS-CoV-2 IgG CLIA. Meaning of symbols: circles = plasma (direct immunoassay), triangles = secondary antibody (indirect immunoassay).

Subsequently, correlation plots were constructed in order to assess the linear relation between the results obtained on the calibrant dilution series with the Surfix method and the reference methods (Figure 8). Very strong, up to near-perfect linear correlations (0.9 < R^2^ < 1) were found when comparing the data from the Surfix direct assay method and all reference methods; the same applies to the Surfix indirect assay method and three of the four (the ELISAs and Abbott CLIA) reference methods, indicating that the dynamic ranges are comparable. Only for the Siemens CLIA method, the correlation was non-linear in the comparison with the Surfix indirect assay method (Figure 8B), which may be related to the higher sensitivity of the Surfix method at lower concentrations (see also Figure 7B).

### 3.3. Testing of the Plasma Verification Panel

**Figure 9:**
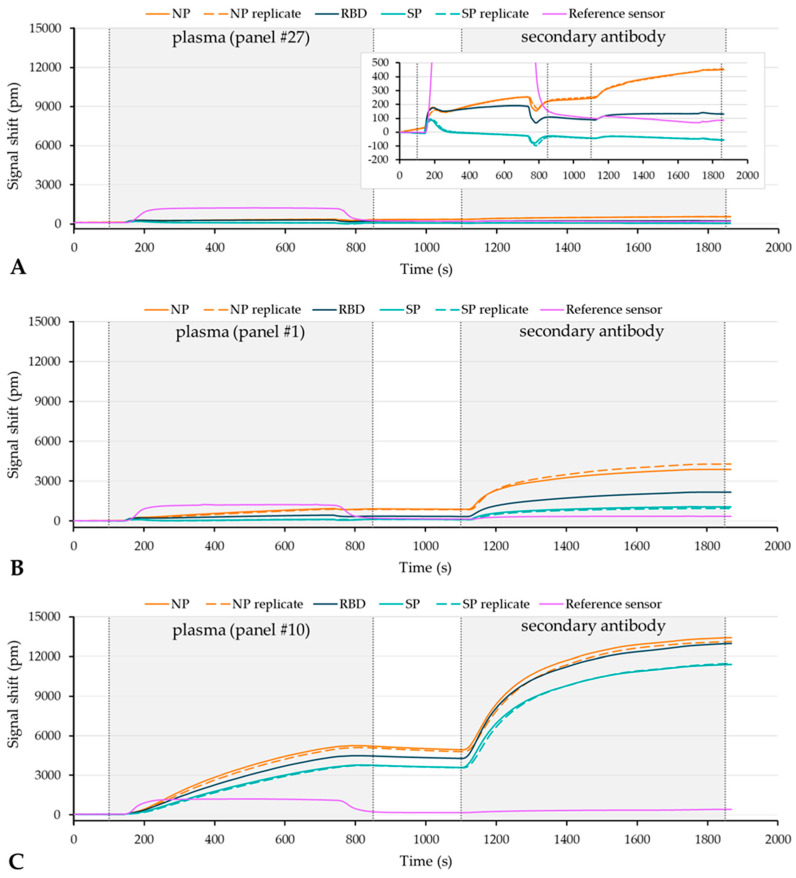
Sensorgrams showing the plasma incubation (direct assay) and the secondary antibody incubation (indirect assay) for (**A**) plasma #27, an anti-SARS-CoV-2 antibody negative plasma (the inset shows a zoom-in), and (**B**) plasma #1, and (**C**) plasma #10, both anti-SARS-CoV-2 antibody positive plasmas.

In a second set of experiments, the diagnostic performance (sensitivity and specificity) of the six Surfix assays was examined by testing 23 anti-SARS-CoV-2 antibody positive and 14 anti-SARS-CoV-2 antibody negative plasma samples of the NIBSC anti-SARS-CoV-2 verification panel. Figure 9 shows a few examples of sensorgrams. Figure 9A is a representative example obtained with one of the negative plasmas (panel #27) exhibiting low but significant binding (signal shift of 100–300 pm) to the immobilized NP and RBD antigens and virtually no binding to the SP antigen (see also the inset). The two positive plasmas (panel #1 (Figure 9B) and #10 (Figure 9C)) both showed substantial binding but on a different overall level and with a different selectivity profile. Plasma #1 showed a clear order in the extent of antibody binding to the different antigens (i.e., NP > RBD > SP), whereas plasma #10 demonstrated a near equal binding outcome for RBD and NP, and a somewhat lower signal for SP. The average binding signal for the three antigens obtained during the incubation with plasma #10 (first incubation step) was approximately 5000 picometer, which corresponds to a calculated binding amount of protein of about 150 ng/cm^2^. The way to calculate the adsorbed surface mass density of protein (ng/cm^2^) from the signal (shift in transmission spectrum in picometer) is explained elsewhere [36]. Assuming that the bound protein consists of head-on oriented IgG (Fc-up; both Fabs bound to the antigen on the surface), a value of 150 ng/cm^2^ represents about half of a monolayer [39]. The ratio between the binding signal that results from the first (plasma) and the second incubation (secondary antibody) is about 1:4 and 1:2 for the sensorgrams in Figure 9B and C, respectively. This signal amplification is in reasonable agreement with the average threefold signal amplification that was found in the calibrant dilution experiments (see above).

**Figure 10:**
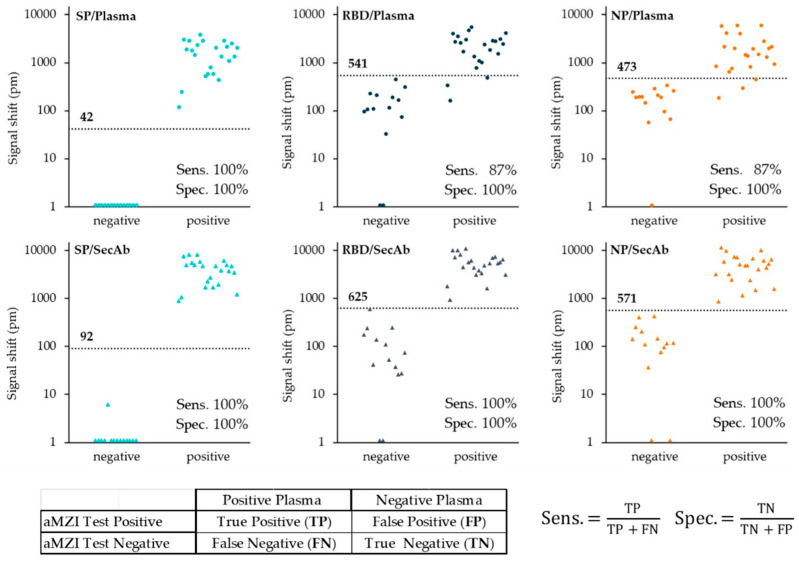
Binding signal distribution of 23 anti-SARS-CoV-2 antibody positive and 14 anti-SARS-CoV-2 antibody negative human plasma samples obtained for the plasma incubation (direct assay: top row) and the secondary antibody incubation (indirect assay: bottom row) shown for the SP (**left**), RBD (**middle**) and NP antigen (**right**). Note 1: for the SP assay, all but one of the negative plasma samples had a small negative binding signal (mean ± standard deviation: −32 ± 13 pm). In order to enable logarithmic presentation all negative values were assigned a value of 1 picometer. Note 2: the small table and the equations at the bottom explains how the sensitivity and specificity was calculated.

A summary of the results obtained with the plasma panel is shown in Figure 10, which shows the binding signal distributions for the plasma incubation (direct assay) and the secondary antibody incubation (indirect assay) as found for the SP, RBD, and NP antigen. What stands out is the excellent performance of the SP assay regarding the distinction between negative and positive samples, which is related to the very low background level of the binding signal as was found for all negative samples. Compared to the SP assay, the RBD and NP assays showed a relatively high and more variable amount of non-specific binding in plasma. At least part of this unwanted background binding signal can be attributed to IgG antibodies since subsequent incubation with the anti-human IgG secondary antibody resulted in a further increased signal. 

Cut-off values were determined as the mean plus three times the standard deviation of the binding signals obtained for all negative plasmas. Based on these cut-off values, diagnostic sensitivity and specificity were calculated for each assay (Figure 10). All assays showed a specificity of 100%. Furthermore, four out of the six assays showed a sensitivity of 100%; only the direct immunoassay with RBD and NP antigen revealed a lower sensitivity of 87%. Please note that these values should be treated with care as the number of samples was limited (23 positive and 14 negative plasmas), which is reflected in the calculated 95% confidence interval (CI) of 85.2–100% and 76.8–100% for the sensitivity and specificity, respectively.

## 4. Discussion

The outcome of this explorative study shows very promising results for the Surfix photonic biosensor platform especially regarding its analytical performance in terms of analytical sensitivity and dynamic range. Furthermore, the measurement results are in accordance with the results obtained by different CE-IVD marked ELISA and CLIA reference methods. The aMZI-based approach is a label-free mass sensing method that very efficiently measures changes in surface mass amounts that come about by the binding of molecules to the sensor surface [40]. Despite the fact that no exogenous labeling (such as chemiluminescence or enzyme labeling) is needed for detection, the added value of the use of a secondary antibody has been clearly demonstrated in the current work by the much improved detection levels of the indirect assay (Table 1). That said, very sensitive detection may not be very relevant for SARS-CoV-2 serology testing but might be relevant for other applications where small analytes or lower clinically relevant concentrations of biomarkers are involved.

The upper limit of the dynamic range of the Surfix assays could not be accurately determined, since no signal saturation was observed at the highest calibrant concentration used (1000 IU/mL, Figure 6). Especially the direct assays are expected to have a very high upper limit. It may therefore be worthwhile to explore the performance of the Surfix assays at higher concentrations of calibrant, since this might be relevant for applications where high antibody titers are expected, for example, in studies concerning vaccine effectiveness. For such applications, the upper limit of currently available assays may be too limited.

Prevention of non-specific binding in the case of plasma sample is challenging because of its high and complex biochemical content. Especially in the case of a direct binding approach, non-specific binding of plasma constituents onto the biosensor surface (fouling) is unwanted, as discrimination between non-specific and specific binding events will be limited [41]. Due to the ease of multiplexing on the photonic biosensor platform, negative control or reference sensors can be easily implemented to compensate for this non-specific binding. Use of an internal reference arm in the balanced aMZI configuration (self-referencing) has an extra advantage as it allows for the direct compensation of the effect from changes in bulk refractive index as can be seen in Figure 9A where the one unbalanced reference sensor exhibits a step-in signal upon introduction of the plasma sample while the other (balanced) sensors show no response. Also shown in Figure 9A is that the SP/BSA balanced aMZI sensor reveals no sign of non-specific binding, but instead, it even shows a slight decrease of signal during incubation with negative control plasma. This is probably explained by a lower degree of non-specific binding that takes place to SP as compared to BSA. In contrast to SP, sensors modified with the RBD and NP antigen did show low but significant non-specific binding when testing the negative controls of the plasma panel (Figure 9A). As a consequence, a poorer discrimination between the anti-SARS-CoV-2 antibody negative and anti-SARS-CoV-2 antibody positive plasma samples was found for the RBD and NP as compared to the SP antigen (Figure 10). The higher level of non-specific binding is very likely attributed to a higher cross-reactivity and/or the higher isoelectric point of the proteins (10.07 for NP and 8.91 for RBD), which makes the proteins positively charged at the near-neutral pH of the assay buffers. Some of the commercial tests for detecting anti-NP antibodies use modified recombinant antigens, for example, the Euroimmun NCP ELISA employs a modified NP that contains only the relevant epitopes in order to prevent background binding. The use of such a truncated NP or other modified viral antigens might also reduce non-specific binding in our NP and/or RBD assay. An alternative to the use of native or recombinant SARS-CoV-2 protein antigen is to employ synthetic peptides that are derived from distinct linear epitope sites of the different SARS-CoV-2 antigens. An increasing number of linear epitopes have been described resulting from immunoinformatic analysis [42], studies with peptide-based ELISA [43] and proteome microarrays [44].

The Surfix photonic biosensor platform enables multiplex detection of antibodies that target viral antigens. The results obtained for three different SARS-CoV-2 antigens (SP, RBD, and NP) were found to correlate well with tests performed with the different CE-IVD marked reference methods (Figure 8). A multiplexing approach opens possibilities for advanced clinical analysis such as: (1) ruling out cross-reactivity with antibodies targeted against other coronaviruses, e.g., the different common cold viruses, to avoid false positive results; (2) simultaneously testing for antibodies against multiple viral antigens may lead to a better reliability of the test; (3) estimating disease severity in COVID-19 patients by using certain peptide epitopes that serve as a disease severity marker [43]; and (4) differentiation and detection of emerging SARS-CoV-2 variants-of-concern.

The presented work shows the potential of the Surfix photonic biosensor for further development into a POC IVD platform. To achieve this, several improvements are currently being implemented. Current developments are focusing on further miniaturization and cost reduction of the photonic chip itself, but also integration of the chip in a microfluidic cartridge to facilitate liquid handling and improve the user-friendliness of the system. Moreover, the read-out instrument and the user interface are being redesigned to meet the requirements of a practical and manufacturable POC IVD platform. In this paper, we have presented results on the sensitive detection of anti-SARS-CoV-2 antibodies in human plasma that target one or more viral antigens. Obviously, by immobilizing different bioreceptors on the sensor waveguides, the aMZI chips can easily be reconfigured for the detection of other targets such as nucleic acids, carbohydrates, viruses, bacteria, as well as small molecules. Hence, the Surfix photonic biosensor is truly a versatile platform technology that can be used in many different application areas. To facilitate the development of devices and applications based on this technology, an R&D system for assay development is being developed in parallel to an IVD system.

## Figures and Tables

**Table 1:** Overview of the blank signals (mean ± standard deviation (SD), *n* = 10) as were found for each of the Surfix assays, and the calculated limits of detection (LODs).

Assay	Mean ± SD (pm)	LOD (IU/mL)
SP/Direct	−66 ± 21	1.4
SP/Indirect	22 ± 10	0.3
RBD/Direct	201 ± 40	9.7
RBD/Indirect	57 ± 25	0.5
NP/Direct	79 ± 34	4.8
NP/Indirect	111 ± 40	2.0

## Data Availability

The authors confirm that the data supporting the findings of this study are available within the article.
